# Primary Meningeal Extraskeletal Osteosarcoma in a Domestic Shorthaired Cat: Case Report and Review of Comparative Pathology

**DOI:** 10.1002/vms3.70771

**Published:** 2026-02-11

**Authors:** Jacqueline Poldy, Dario Costanza, Aran Nagendran, Albert Aguilera‐Padros, Tiziana Liuti, Jorge Del‐Pozo

**Affiliations:** ^1^ Royal (Dick) School of Veterinary Studies Veterinary Pathology Unit the University of Edinburgh, Easter Bush Campus Midlothian UK; ^2^ Interdepartmental Centre of Veterinary Radiology University of Napoli “Federico II” Napoli Italy; ^3^ Royal (Dick) School of Veterinary Studies Hospital for Small Animals the University of Edinburgh, Easter Bush Campus Midlothian UK

**Keywords:** feline, meninges, MRI, osteosarcoma

## Abstract

This case describes the presentation and diagnosis of an extraskeletal osteosarcoma arising in the cranial meninges of a domestic shorthaired cat. Clinical signs were compatible with a space‐occupying cerebral lesion, and magnetic resonance imaging revealed a multilobular mass with extracranial and intracranial components, which asserted marked forebrain mass effect and apparently originated from the frontal and parietal bones. Due to clinical deterioration and poor prognosis, the cat was euthanized and a post‐mortem examination performed. Gross lesions confirmed a solitary neoplasm, firmly attached to the dura mater, overlying and compressing the left cerebral hemisphere. The neoplasm was not adherent to bony structures of the skull, but local osteolytic destruction of the frontal bone and cribriform plate allowed its extension outside the calvarium and into the nasal sinuses. The tissue was diagnosed by histological assessment as an osteosarcoma. Primary extraskeletal osteosarcomas of the meninges are exceedingly rare in all species, and to the authors’ knowledge, have never been reported in cats. As the clinical behaviour is very different from more typically encountered neoplasms, this possibility, although rare, may be considered as a differential diagnosis for feline intracranial tumours.

## Graphical abstract

A domestic shorthaired cat with neurological signs compatible with a forebrain lesion was investigated with magnetic resonance imaging, revealing a mass with intracranial and extracranial components. Due to the poor prognosis the cat was euthanized, and gross and histopathological examination identified an extraskeletal osteosarcoma with its origin in the meninges. This is the first report of a primary meningeal osteosarcoma in a cat, and the imaging and pathology findings are compared with documented cases in other species.

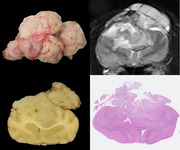

## 1 Introduction

Osteosarcomas are composed of malignant mesenchymal osteoid‐producing cells, which most commonly arise from medullary bone (central osteosarcomas) affecting the appendicular and, less frequently, the axial skeleton. Osteosarcomas are the most common primary tumours of bone in cats and dogs. With an incidence of approximately three to five per 100,000 animals (Dorn et al. 1968), central and surface (juxtacortical) osteosarcomas comprise approximately 80% to 95% of primary bone tumours in cats (Liu et al. 1974; Quigley and Leedale 1983). The prevalence in dogs is approximately 10‐fold higher, with a UK study reporting a 1‐year prevalence of 331 cases from 905,552 dogs (0.037%) (O'Neill et al. 2023). Feline central osteosarcomas tend to have a lower metastatic potential with respect to their canine counterparts (Dimopoulou et al. 2008).

Osteosarcomas may also arise from inducible osteogenic precursor cells within soft tissues, giving rise to a primary neoplasm not associated with skeletal structures. These extraskeletal/extraosseous osteosarcomas are considered to be extremely rare presentations, although a paucity of studies and low absolute numbers may limit the reliability of such comparisons. Reports of their prevalence vary considerably (Patnaik 1990; Kuntz et al. 1998; Langenbach et al. 1998); in one study of 145 feline osteosarcomas, around 40% were reported to be of extraskeletal origin (Heldmann et al. 2000). These tend to behave more aggressively and carry a worse prognosis than central neoplasms. The majority originate in subcutaneous tissues, frequently at sites associated with injected vaccinations. Other sporadically reported sites include mammary (Heldmann et al. 2000), orbital/ocular (Woog et al. 1983; Peiffer et al. 1988; Dubielzig et al. 1990; Heldmann et al. 2000; Groskopf et al. 2010), oral (Heldmann et al. 2000), hepatic (Dhaliwal et al. 2003) and intestinal/omental (Lean et al. 2025; Stimson et al. 2000). Among primary extraskeletal osteosarcomas, meningeal origins are exceedingly rare in dogs, and there are no published records in cats.

Magnetic resonance imaging (MRI) is the gold standard imaging modality to assess the brain, and skull bone neoplasia is occasionally detected (Saunders and Boroffka 2018). Aggressive tumours such as osteosarcoma and chondrosarcoma tend to cause extensive lysis of the skull bones, potentially penetrating the calvarium and leading to neurological clinical signs due to compression of cerebral structures (Tam et al. 2022). On MRI, these lesions exhibit irregular signal intensity and variable contrast enhancement. Signal voids are often present, corresponding to residual destroyed bone, new bone formation or areas of mineralisation. While studies in veterinary literature describe the MRI features of cranial osteosarcoma (Tam et al. 2022), no reports detail the MRI characteristics of intracranial meningeal osteosarcomas. In human literature, limited reports describe these tumours as compressive lesions with heterogeneous iso‐ to hypointense signals on T1‐weighted (T1W) images, and iso‐ to hyperintense signals on T2‐weighted (T2W) images. They are irregular, predominantly peripheral contrast‐enhancing, with an associated marked mass effect on the surrounding brain parenchyma (Zhang et al. 2016; Chen et al. 2016). In this report, we describe the clinical presentation, MRI findings and histopathological findings of a feline meningeal osteosarcoma.

## 2 Case Summary

A 12‐year‐old, neutered male domestic shorthaired cat, weighing 4.6 kg, was presented as an emergency referral on account of lethargy, compulsive pacing, circling and progression to marked altered mentation, which had commenced 7 days prior. Non‐steroidal anti‐inflammatory drugs (meloxicam, 0.05 mg/kg PO, once a day for 5 days) had been initiated by the referring veterinarian, with a suspicion of osteoarthritic pain, but clinical signs had progressed to obtundation, reduced appetite, involuntary faecal and urinary elimination and circling. A slight bump was noted on the cat's head by the owner. There was no history of trauma and no apparent pain, although the owner had observed a small amount of blood splatter on bedding and suspected its source via sneezing. The cat was housed with another healthy cat, mainly indoors, was fully vaccinated and had no previous health concerns.

Upon presentation, all vital parameters were within normal limits, but the cat was obtunded and displayed wide circling in both directions, compulsive pacing and mild proprioceptive ataxia in all four limbs and tetraparesis. Postural reactions, spinal reflexes and cranial nerve examination were normal. A hard (bone‐like), non‐painful, small lump was palpable in the left frontal area. Neurological localisation was compatible with a forebrain lesion, for which differential considerations were a neoplastic process, an inflammatory/infectious disease (e.g., toxoplasmosis or meningoencephalitis of unknown origin) with a suspected secondary increased intracranial pressure. Lastly, vascular injury (cerebrovascular accident) was considered, but less likely due to the prolonged progression of signs. Haematology and serum biochemistry performed by the referring veterinarian showed a mild increase in creatine kinase (652.7 U/L, reference: 20.0–225.0) and cholesterol (5.16 mmol/L, reference: 2.20–4.00), with other parameters being unremarkable. Serum T4 was within the reference range.

MRI of the head was performed under general anaesthesia using a high‐field 1.5T unit (Siemens Magnetom Avanto, Erlangen, Germany) equipped with a 15‐channel transmit/receive coil (model Tx/Rx 15‐Channel Knee coil). The adopted institutional protocol included dorsal, sagittal and transverse T2W, transverse fluid‐attenuated inversion recovery (T2W‐FLAIR), transverse T2W gradient echo (T2*W‐GE), transverse T1W, diffusion‐weighted imaging (DWI) and apparent diffusion coefficient (ADC) sequences. After contrast administration (0.1 mmol/kg of gadoteridol IV; Prohance, Bracco Diagnostics, Milan, Italy), transverse T1W and three‐dimensional gradient‐echo sequences (T1W MP‐RAGE) were also acquired. All the sequences were acquired with a slice thickness of 2.5 mm except the MP‐RAGE, where a 0.8 mm slice thickness was used.

**FIGURE 1:**
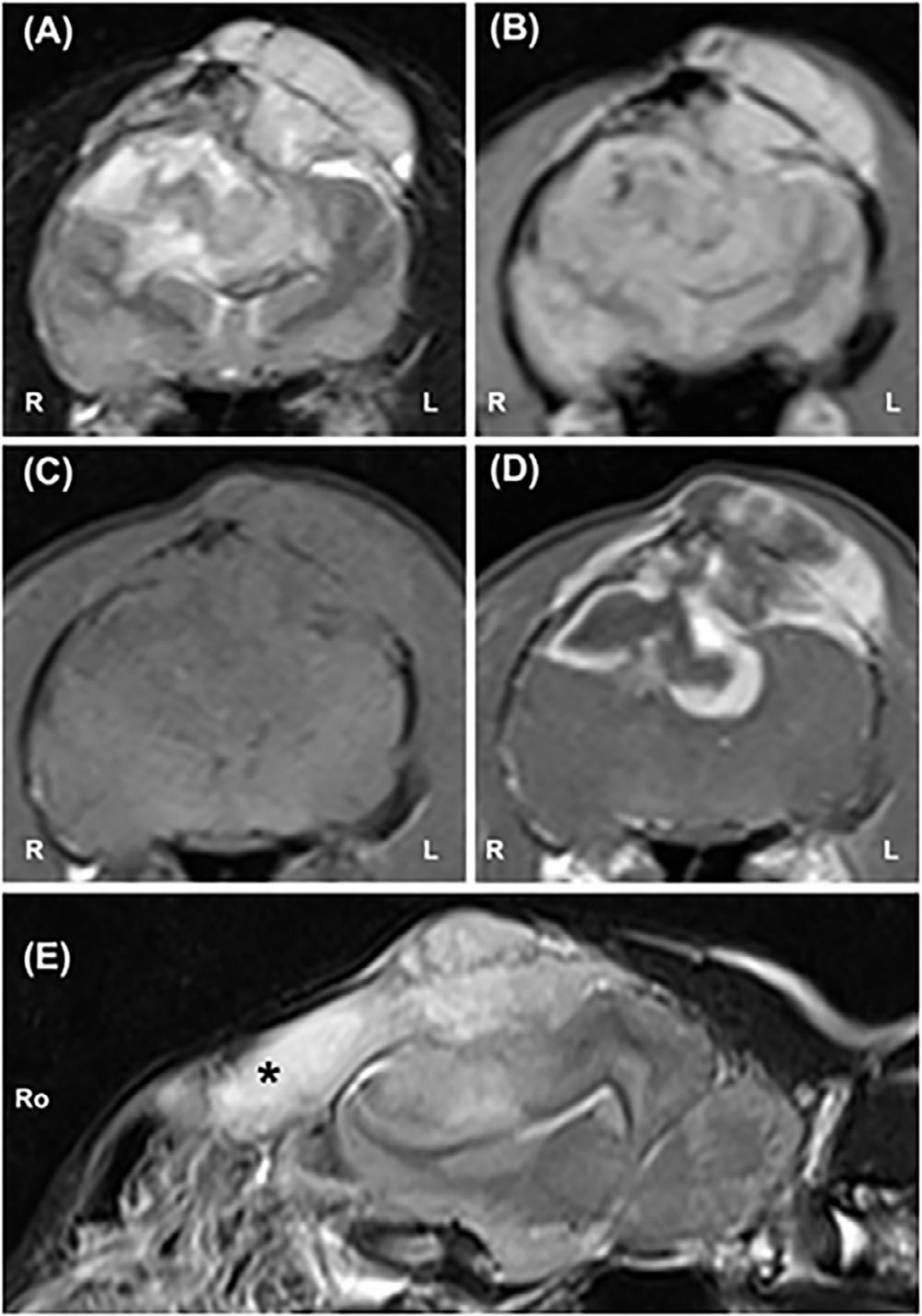
T2W (A), T2*W‐GE (B), T1W precontrast (C) and T1W postcontrast (D) transverse MR images at the level of the parietal bones, and T2W left‐parasagittal MR image of the skull. A large, amorphous lesion, diffusely T2W hyperintense (A) with multiple small signal voids in the T2*W‐GE sequence (B), involving both the left and right cerebral hemispheres, causing sinistrad displacement of the falx cerebri and marked compression of both lateral ventricles, is visible. In the precontrast T1W image (C), the multiple parietal bone lytic defects caused by the mass are best appreciated. After contrast administration, the mass is markedly, mainly peripherally contrast‐enhancing (D). In the sagittal T2W image (E), the mass effect of the lesion on the cerebrum and cerebellum is readily appreciated, as well as the invasion of the left frontal sinus (black asterisk). L, left; R, right; Ro, rostral.

The MRI revealed a large, amorphous mass lesion with intracranial and extracranial components (Figure 1). The intracranial portion of the lesion was diffusely T2W hyperintense (Figure 1A), partially lined by a dural tail and occupied a large dorsorostral area of the cranial cavity. It extended from the left olfactory lobe caudally, infiltrating both parietal lobes’ parenchyma and reaching the internal capsule. Within the intracranial portion of the mass, the T2*W‐GE sequence revealed multiple small signal voids likely representing microbleeding or mineralization (Figure 1B). The mass was mainly T1W iso‐ and hypointense compared to the normal grey matter (Figure 1C) and displayed marked, although irregular, mainly peripheral contrast enhancement (Figure 1D). The lesion exerted a significant mass effect on the brain parenchyma, causing caudo‐ventral and leftward compression of the forebrain (Figure 1E), with both subfalcine and transtentorial herniation. The cerebellum was also minimally displaced caudally, with apparent partial herniation through the foramen magnum.

The extracranial portion of the lesion, with a maximum thickness of 6 mm, passed through multiple lytic permeative lesions affecting both parietal and temporal bones, best appreciated in T1W sequences (Figure 1C). A rostral extension of the mass was also present within the left frontal sinus, which was entirely effaced by the mass (Figure 1E, black asterisk). The DWI images showed increased signals on ADC maps, indicating increased diffusion likely due to vasogenic oedema. The most likely imaging diagnosis was an extra‐cranial neoplastic mass originating from the frontal and parietal bones, primarily compressing the forebrain. Primary differentials included chondrosarcoma, osteosarcoma or squamous cell carcinoma. Less likely considerations were primary extra‐axial brain lesions such as meningioma or lymphoma with secondary involvement of the calvarial bones.

Due to the extent and invasion of the mass, surgical and radiotherapeutic management were discussed but considered unfeasible for the patient, and the owner elected for euthanasia. A post‐mortem examination was performed the following day.

**FIGURE 2:**
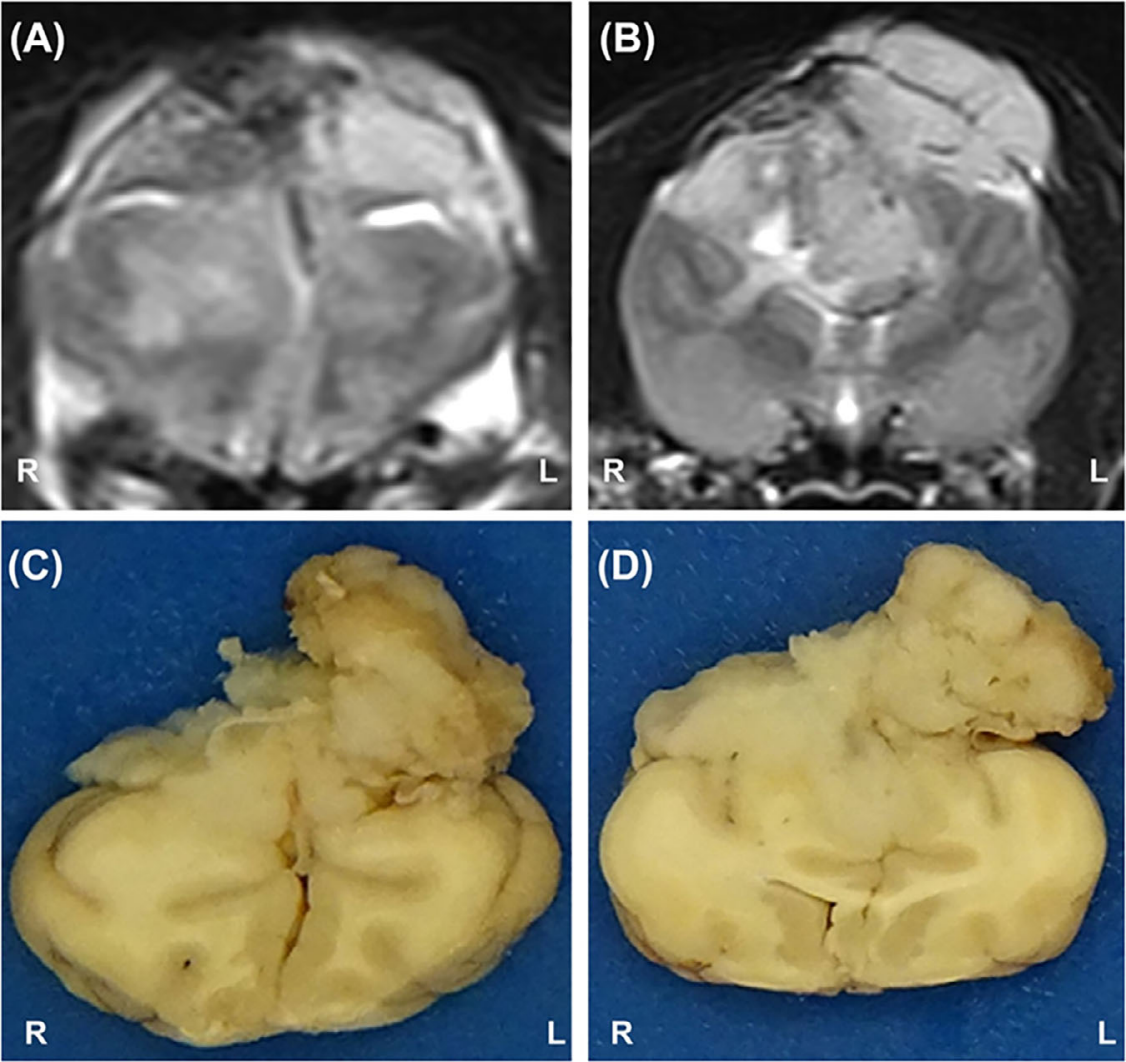
T2W MR images (A, B) and corresponding brain transverse sections (C, D) at the level of the frontal (A, C) and parietal lobes (B, D). The frontal lobes (A, C) and the cerebral hemispheres (B, D) are markedly compressed, distorted and partially infiltrated by the mass that has an extracranial component and extends within the parenchyma up to the internal capsule. L, left; R, right.

On gross examination, overlying the dorsal cerebral hemispheres was a firm, multilobular, expansile, off‐white to pale pink mass, measuring approximately 5.0 × 3.5 × 1.2 cm. The tissue pierced the overlying cranium at the dorsal midline, extending over the left parietal bone and spread rostrally through the cribriform plate to entirely occlude the left nasal sinus. The mass was firmly adherent to the cerebral dura mater and incorporated the rostral falx cerebri. There was no attachment to the bony structures through which it penetrated. No other appendicular or axial changes were identified. In transverse sections, the cerebral hemispheres were markedly compressed and partially infiltrated by the mass, from the frontal lobes to the level of the internal capsule (Figure 2).

**FIGURE 3:**
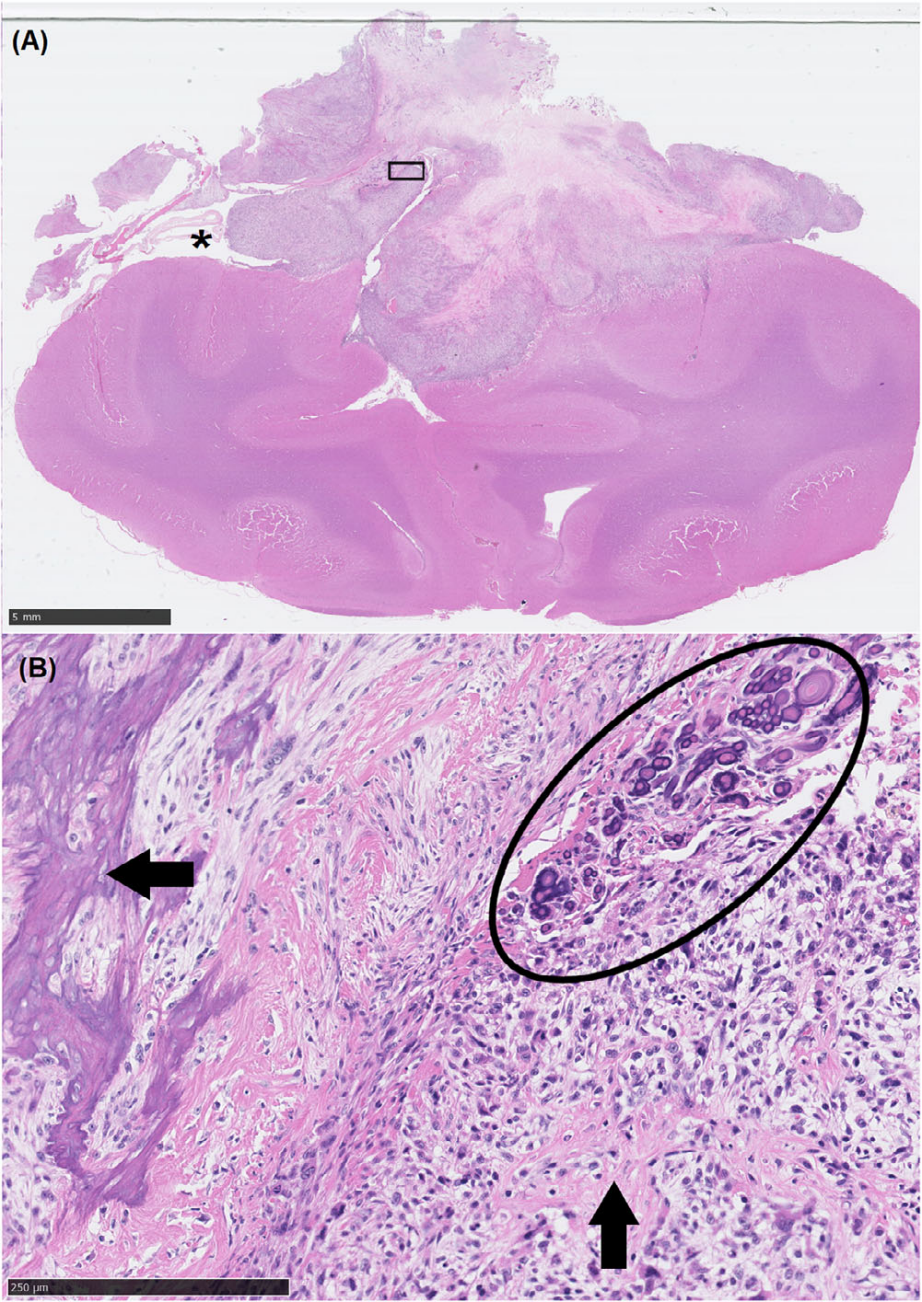
Histological sections of the brain and meningeal mass. (A) A transverse section through the frontal lobes (corresponding to the levels indicated in Figure 2A,C) shows the compressive and partially infiltrative nature of the neoplasm. A portion of entrapped meningeal tissue is visible (asterisks). Haematoxylin and eosin (H&E). Scale bar = 5 mm. (B) Higher magnification of the selected rectangular area from (A) reveals haphazard streams of pleomorphic polygonal to spindleoid neoplastic cells, producing abundant osteoid (arrows), which is partially mineralized at the right of the image (horizontal arrow). Clusters of psammoma body concretions are present in the top right of the field of view (ellipse). H&E. Scale bar = 250 µm.

Tissues were fixed in 10% neutral buffered formalin, decalcified with Decalcifier I solution (Leica, UK), embedded in paraffin, and sectioned at 5 µm according to standard procedures. Sections were stained with haematoxylin and eosin for histological evaluation. Microscopically (Figure 3), the mass was composed of a pleomorphic proliferation of polygonal to spindle‐shaped cells, arranged in fasciculating bundles and storiform streams, incorporating elements of pre‐existing meninges. The cells had moderate amounts of fibrillary eosinophilic cytoplasm, poorly delineated cell borders and round to elongate nuclei with coarsely stippled to vesicular chromatin and large prominent nucleoli. Mitoses averaged 30 in 2.37 mm^2^, including several bizarre mitoses. Multifocally, the cells surrounded and were embedded within irregular lakes of fibrillary to lamellar matrix (osteoid), which ranged from pale to brightly eosinophilic to variable basophilic (mineralisation). Aggregates of deeply basophilic concentric mineralized concretions (psammoma bodies) were also present in isolated foci. A second population of large, irregularly polygonal, multinucleate cells (osteoclasts) was variably present throughout the neoplasm. There were large areas of necrosis and scattered lymphocytic inflammation throughout the mass. At the periphery, local clusters of neoplastic cells infiltrated the compressed cortical parenchyma. A diagnosis of meningeal osteosarcoma was reached.

Additional histological changes included acute thromboembolism of multiple small‐calibre pulmonary vessels. Mild chronic lymphocytic interstitial nephritis and hepatitis, nodular pancreatic hyperplasia and mild islet amyloidosis were also present. These latter conditions are common and generally incidental age‐related changes in cats and were considered not to have been clinically significant.

## 3 Discussion

Intracranial neoplasia in cats is reported to have an incidence of 3.5 per 100,000 (0.0035%) to 2%, with meningiomas representing the large majority (58%) (Troxel et al. 2003; Rissi 2023). Meningiomas are extra‐axial neoplasms that arise from arachnoid cap cells of the meninges (Rissi 2023), often associated with arachnoid granulations within the dural venous sinuses. Although they are generally benign neoplasms and usually do not infiltrate the brain, their expansile nature compresses the neural tissue. An evaluation of the clinicopathological features of cats with T1–T6 myelopathies assessed by MRI and/or CT found that of neoplastic disease in the thoracic vertebral region, meningioma and lymphoma occurred with equal frequency. The authors highlight the similar imaging features exhibited by both tumours (intradural‐extramedullary) (Benito et al. 2023). In this case, although a meningioma was considered a differential diagnosis, its likelihood was reduced given the MRI and gross presentation characterised by the extensive lysis of the skull bones and the infiltrative behaviour of the lesion (Troxel et al. 2004). In addition, the destruction of the cribriform plate and invasion of the frontal sinuses are atypical for meningiomas (Pérez‐Accino et al. 2019). This differential was excluded upon histologic examination, which revealed a tumour architecture, cellular morphology and abundant osteoid, indicative of osteosarcoma. Of the six histological variants, the most frequent subtypes in cats are osteoblastic, fibroblastic, chondroblastic and telangiectatic (Negrin et al. 2006). In the present case, variably abundant irregular islands of osteoid are consistent with an osteoblastic classification.

In cats, osteosarcomas involving the skull are rare (Negrin et al. 2006). In this case, an aggressive extra‐axial lesion―such as a skull bone osteosarcoma or chondrosarcoma―was considered as the primary differential based on the MRI findings, including the extensive lysis of the skull bones with invasion into the cranial cavity and secondary mass effect on the encephalon, the irregular signal intensity and contrast enhancement, and the signal voids visible in the T2*W‐GE images suggesting areas of mineralisation or microbleeding in the mass. However, post‐mortem examination revealed that the mass was firmly adherent to the meninges but distinctly unattached to the dorsal calvarium, indicating an extraskeletal origin. Primary meningeal osteosarcomas are exceeding rare entities and have not previously been described in cats. Feline extraskeletal osteosarcomas occur most commonly in subcutaneous tissues at sites associated with vaccination, and with much lower frequency, in other tissues. A history of trauma has been suggested as a predisposing factor in the development of orbital and ocular osteosarcomas (Woog et al. 1983; Peiffer et al. 1988; Dubielzig et al. 1990).

Primary meningeal osteosarcomas are best documented in the human literature, but even here, cases are infrequent and heterogeneous. Among the 31 published records in humans (Table 1), there is an equal sex distribution and a wide age range, which has an asymmetric bimodal distribution. Six cases (19%) are documented in children (≤ 16 years old), and frequencies peak in men and women in their 50s and 60s (55%), after a nadir during early adulthood to middle‐age. Among older patients, radiotherapeutic interventions from 7 months to 24 years previously appeared to play a role in the induction of osteosarcoma at the site of a former, unrelated neoplasm (Couldwell et al. 1997; Osipov et al. 2002; Sanno et al. 2004; Ziewacz et al. 2010; Horan et al. 2021). In the veterinary literature, meningeal osteosarcomas have been described on four occasions in dogs (Ringenberg et al. 2000; Bonanno et al. 2019; Rosolem et al. 2021; Riggers et al. 2022), and once in a rat (Pace et al. 1995). In none of these cases was there any history of carcinogenic induction through radiation or other means, and they were considered spontaneous neoplasms.

**TABLE 1:** Comparative pathological of primary meningeal extraskeletal osteosarcomas.

Species	Sex	Age (years)	Location, origin	Features	Reference
H	F	3	Right temporal, A		Bauman et al. (1997)
H	M	3	Left temporoparietal, A		Pourrashidi Boshrabadi et al. (2017)
H	F	6	Left hemisphere, D		Zhang et al. (2016)
H	M	8	Right frontotemporal, A		Dagcinar et al. (2008)
H	F	16	Left temporal, D		Alleyne et al. (2000)
H	F	16	Left parietal, A		Sipos et al. (1997)
H	F	37	Left frontoparietal, D		Turner and Craig (1941)
H	F	42	Parasagittal frontoparietal, D		Bar‐Sela et al. (2001)
H	F	43	Right frontotemporal, D		Ghosal et al. (2010)
H	M	45	Pineal region, A		Saesue et al. (2004)
H	F	46	Falx, right frontal, D	RT	Horan et al. (2021)
H	F	49	Left frontal, A	OCSA/GBM	Sarmiento et al. (1979)
H	M	51	Left frontal, D	RT	Osipov et al. (2002)
H	M	51	Left frontotemporal, D		Romeo et al. (2010)
H	F	53	Right frontoparietal, D	RT	Sanno et al. (2004)
H	M	53	Right temporoparietal, A		Jacques et al. (1976)
H	M	54	Left frontoparietal, D		Qiu and Chen (2021)
H	F	54	Left frontal, A		Chen et al. (2016)
H	M	54	Tentorium cerebelli, A	RT, Mets	Ziewacz et al. (2010)
H	M	56	Falx, bifrontal, D		Wang et al. (1986)
H	M	56	Left temporal, D	RT	Couldwell et al. (1997)
H	M	56	Left frontodorsal, D		Setzer et al. (2002)
H	F	57	Right arietal, A		Ohara et al. (1994)
H	F	64	Left frontal, D		Lam et al. (1981)
H	M	64	Right falx, bifrontoparietal, D		Zirh et al. (1992)
H	M	64	Right thalamic, A		Reznik and Lenelle (1991)
H	F	64	Cerebellar, A	Epidermoid	Cannon et al. (1998)
H	F	68	Sacral spine, D	CA	Pal et al. (2008)
H	F	70	Spine, D	CA	Schiller et al. (2013)
H	M	74	Left frontotemporal, A		Walker et al. (2001)
H	M	78	Left frontal, A		Hettmer et al. (2002)
C, mixed breed	M	8	Sella turcica, D		Rosolem et al. (2021)
C, Labrador	F	9.5	C3‐C4 spine, D		Riggers et al. (2022)
C, English Pointer	MN	10	Left cerebellum, D		Ringenberg et al. (2000)
C, mixed breed	M	11	C6‐C7 spine, D		Bonanno et al. (2019)
R, albino	F	2	Left parietal, D		Pace et al. (1995)
Fe, DSH	MN	12.5	Left frontoparietal, D		Current report

Abbreviations: A, entirely intra‐axial; C, canine; CA, previous oil‐based contrast agents for myelography; D, dura based; DSH, domestic shorthair; epidermoid, associated with epidermoid lesion; F, female; Fe, feline; H, human; M, male; Mets, developed thoracolumbar spinal metastases; MN, male neutered; OCSA/GBM, cerebral mixed tumour: osteo‐chondrosarcoma/glioblastoma multiforme; R, rat; RT, previous radiotherapy.

All canine cases involved middle‐aged to older dogs, between 8 and 11 years old, with no breed predilection (Table 1). Two were cranial lesions involving the meninges of the cerebellum (Ringenberg et al. 2000), and the skull base associated with the sella turcica (Rosolem et al. 2021), and two originated in the dura mater of the cervical spine (Bonanno et al. 2019; Riggers et al. 2022). Although low numbers caution against over‐generalisation, this anatomic distribution (1:1 intracranial to spinal) differs from that reported in humans, in which only two of the 31 cases comprised a primary spinal presentation. In both human cases, oil‐based contrast agents had been used during myelography for investigation of unrelated conditions (sciatic myalgia and childhood meningitis), several decades prior to the development of osteosarcoma in the lumbar and sacral spinal canal (Pal et al. 2008; Schiller et al. 2013). Myelographic agents are known to be associated with dural osseous metaplasia and, in at least one of these cases, the neoplastic tissue arose within such an area, suggesting malignant transformation.

In the two cranial canine cases, (Ringenberg et al. 2000; Rosolem et al. 2021) only the anatomopathological findings were reported, with no advanced imaging techniques utilized. To our knowledge, this is the first case report in veterinary medicine describing the MRI findings of a primary intracranial meningeal osteosarcoma. The lesion exhibits imaging characteristics similar to those reported for skull osteosarcoma in both dogs and cats (Tam et al. 2022), making distinguishing between the two types challenging. In addition, these imaging features resemble those described in the few documented cases of primary meningeal osteosarcoma in human patients (Zhang et al. 2016; Chen et al. 2016).

In two of the canine cases, the osteosarcomas originated within sites of ossifying pachymeningitis (Ringenberg et al. 2000; Rosolem et al. 2021). These focal deposits of mineral and ectopic bone are common, usually incidental changes that arise within the cranial or spinal dura mater of aged dogs. Whether there exists a sequential progression from mineralization to ectopic ossification, and with ultimate neoplastic transformation, is difficult to say. Such transformation is at least conceivable, and in line with occurrence of dural osteosarcoma in human cases at sites of myelography‐induced osseous metaplasia (Schiller et al. 2013). In vitro studies have found mitogenic activity and colony forming efficiency to be higher for osteoblasts from sites of heterotopic ossification compared to normal marrow‐derived osteoblasts (Sell et al. 1998), suggesting that disparate behaviour may offer fertile grounds for neoplastic transformation.

Dagcinar et al., in reviewing the literature of primary meningeal osteosarcomas in humans to 2008 (at that time, 20 cases), distinguished tumours that arose within the dura mater from those with an entirely intra‐axial localisation (Dagcinar et al. 2008). The authors assert a better prognosis for the former group, and suggest that this may relate to the efficacy of total excision for superficial tumours compared with intra‐cerebral. All the veterinary cases involve lesions originating within the dura. In two canine cases, surgical treatment was attempted. One case failed to recover successfully, suffering multiple, ultimately terminal cardiopulmonary arrests (Riggers et al. 2022). The other showed clinical improvement following surgery and post‐surgical chemotherapy, before acutely deteriorating 2 months postoperatively (Bonanno et al. 2019).

Histologic assessment of pulmonary tissue from this cat revealed numerous microthrombi within the pulmonary microvasculature. Although pulmonary thromboembolism (PTE) can be a serious, potentially life‐threatening condition, small thromboemboli may be of no functional significance and it is rarely recognized clinically in cats (Norris et al. 1999). Indeed, there had been no clinically apparent respiratory compromise in this individual. Diverse risk factors and disease entities have been associated with PTE through variable perturbations of Virchow's triad—hypercoagulability, vascular flow anomalies and/or endothelial injury (Goggs et al. 2009). Malignancy‐associated coagulopathy (MAC) is a well‐recognized hypercoagulable state that may increase the risk of thromboembolic complications in cancer patients. The pathogenesis of MAC is believed to be due to platelet activation, initiation of the coagulation cascade and suppression of fibrinolysis. Recent evaluation of haemostatic parameters in human patients with bone tumours, indicated a hypercoagulable profile in these patients. Coagulability was due to rapid initiation of the coagulation cascade stimulated by tumour‐derived factors, and platelet activation (Tsantes et al. 2022). In cats, when PTE is documented secondary to neoplastic conditions (Norris et al. 1999), MAC presumably plays a role in its pathogenesis. In this individual, no other significant pathological conditions that might be considered procoagulant were identified.

## 4 Conclusion

This report documents ante‐mortem, advanced imaging and post‐mortem macroscopic and histologic features of a meningeal osteosarcoma in a cat. The gross and histologic involvement of the dura mater within the neoplasm, and the absence of gross changes in the appendicular or axial skeleton, were consistent with a primary meningeal lesion. This is the first report of a meningeal osteosarcoma in a cat, and resembles similar presentations to those reported in humans, dogs and a rat.

## Author Contributions

**Jacqueline Poldy**: conceptualization, investigation, writing – original draft, writing – review and editing. **Dario Costanza**: investigation, writing – review and editing. **Aran Nagendran**: investigation, writing – review and editing. **Albert Aguilera Padros**: investigation, writing – review and editing. **Tiziana Liuti**: supervision, writing – review and editing, conceptualization. **Jorge Del‐pozo**: supervision, writing – review and editing, conceptualization.

## Funding

The authors have nothing to report.

## Ethics Statement

The authors confirm that the ethical policies of the journal, as noted on the journal's author guidelines page, have been adhered to. No ethical approval was required as this is a single case report detailing a clinical case managed in keeping with the RCVS (Royal College of Veterinary Surgeons) professional guidelines and did not involve research procedures.

## Conflicts of Interest

The authors declare no conflicts of interest.

## Data Availability

The authors have nothing to report.

## References

[vms370771-bib-0001] Alleyne, C. H., Jr. , N. Theodore , R. F. Spetzler , and S. W. Coons . 2000. “Osteosarcoma of the Temporal Fossa With Hemorrhagic Presentation: Case Report.” Neurosurgery 47: 447–451. 10942019 10.1097/00006123-200008000-00036

[vms370771-bib-0002] Bar‐Sela, G. , T. Tzuk‐Shina , M. Zaaroor , Y. Vlodarsky , M. Tsalik , and A. Kuten . 2001. “Primary Osteogenic Sarcoma Arising From the Dura Mater: Case Report.” American Journal of Clinical Oncology 24: 418–420. 11474278 10.1097/00000421-200108000-00025

[vms370771-bib-0003] Bauman, G. S. , W. M. Wara , S. F. Ciricillo , R. L. Davis , S. Zoger , and M. S. B. Edwards . 1997. “Primary Intracerebral Osteosarcoma: A Case Report.” Journal of Neuro‐Oncology 32: 209–213. 9049882 10.1023/a:1005756930169

[vms370771-bib-0004] Benito Benito, M. , B. A. Lopes , R. José‐López , et al. 2023. “Demographics, Clinical Findings and Diagnoses of Cranial Thoracic Myelopathies (T1–T6 vertebrae) in Cats.” Journal of Feline Medicine and Surgery 25: 1098612X231199731. 10.1177/1098612X231199731 PMC10812017 37791892

[vms370771-bib-0005] Bonanno, G. , S. A. Gomes , S. Dawson , J. Bass , and M. Lowrie . 2019. “Intradural‐Extramedullary Osteosarcoma in a Dog.” Veterinary Record Case Reports 7: e000770.

[vms370771-bib-0006] Cannon, T. C. , B. L. Bane , D. Kistler , and G. W. Schoenhals . 1998. “Primary Intracerebellar Osteosarcoma Arising Within an Epidermoid Cyst.” Archives of Pathology & Laboratory Medicine 122: 737–739. 9701337

[vms370771-bib-0007] Chen, S. P. , J. L. Tang , and X. L. Zhu . 2016. “Primary Intracerebral Osteosarcoma: A Rare Case Report and Review.” SpringerPlus 5: 1997. 27933253 10.1186/s40064-016-3678-z PMC5118371

[vms370771-bib-0008] Couldwell, W. T. , B. W. Scheithauer , S. G. Rice , W. Zhang , and C. B. Stillerman . 1997. “Osteosarcoma of the meninges in Association With Glioblastoma.” Acta Neurochirurgica 139: 684–689. 9265963 10.1007/BF01412005

[vms370771-bib-0009] Dagcinar, A. , F. Bayrakli , O. Yapicier , and M. Ozek . 2008. “Primary Meningeal Osteosarcoma of the Brain During Childhood: Case Report.” Journal of Neurosurgery: Pediatrics 1: 325–329. 18377310 10.3171/PED/2008/1/4/325

[vms370771-bib-0010] Dhaliwal, R. S. , T. O. Johnson , and B. E. Kitchell . 2003. “Primary Extraskeletal Hepatic Osteosarcoma in a Cat.” Journal of the American Veterinary Medical Association 222: 340–342. 12564597 10.2460/javma.2003.222.340

[vms370771-bib-0011] Dimopoulou, M. , J. Kirpensteijn , H. Moens , and M. Kik . 2008. “Histologic Prognosticators in Feline Osteosarcoma: A Comparison With Phenotypically Similar Canine Osteosarcoma.” Veterinary Surgery 37: 466–471. 18986314 10.1111/j.1532-950X.2008.00409.x

[vms370771-bib-0012] Dorn, C. R. , D. O. N. Taylor , F. L. Frye , and H. H. Hibbard . 1968. “Survey of Animal Neoplasms in Alameda and Contra Costa Counties, California. I. Methodology and Description of Cases.” JNCI: Journal of the National Cancer Institute 40: 295–305. 5694271

[vms370771-bib-0013] Dubielzig, R. R. , J. Everitt , J. A. Shadduck , and D. M. Albert . 1990. “Clinical and Morphologic Features of Post‐Traumatic Ocular Sarcomas in Cats.” Veterinary Pathology 27: 62–65. 2309385 10.1177/030098589002700111

[vms370771-bib-0014] Ghosal, N. , R. Dadlani , S. V. Furtado , N. Bagdi , and A. S. Hegde . 2010. “Dural Based Primary Osteosarcoma in Right Fronto‐Temporal Region With Review of Literature.” Neurology India 58: 128–130. 20228482 10.4103/0028-3886.60428

[vms370771-bib-0015] Goggs, R. , L. Benigni , V. L. Fuentes , and D. L. Chan . 2009. “Pulmonary Thromboembolism.” Journal of Veterinary Emergency and Critical Care 19: 30–52. 19691584 10.1111/j.1476-4431.2009.00388.x

[vms370771-bib-0016] Groskopf, B. S. , R. R. Dubielzig , and S. L. Beaumont . 2010. “Orbital Extraskeletal Osteosarcoma Following Enucleation in a Cat: A Case Report.” Veterinary Ophthalmology 13: 179–183. 20500718 10.1111/j.1463-5224.2010.00774.x

[vms370771-bib-0017] Heldmann, E. , M. A. Anderson , and C. Wagner‐Mann . 2000. “Feline Osteosarcoma: 145 Cases (1990‐1995).” Journal of the American Animal Hospital Association 36: 518–521. 11105889 10.5326/15473317-36-6-518

[vms370771-bib-0018] Hettmer, S. , G. Fleischhack , C. Hasan , T. Kral , B. Meyer , and U. Bode . 2002. “Intracranial Manifestation of Osteosarcoma.” Pediatric Hematology and Oncology 19: 347–354. 12078866 10.1080/08880010290057363

[vms370771-bib-0019] Horan, S. , O. Breathnach , L. Grogan , et al. 2021. “A Rare Case of High‐Grade Chondroblastic Osteosarcoma Post‐Radiotherapy for a Right Frontal Oligodendroglioma.” Journal of Clinical Images and Medical Case Reports 2: 1033.

[vms370771-bib-0020] Jacques, S. , D. B. Freshwater , and C. H. Shelden . 1976. “Primary Osteogenic Sarcoma of the Brain: Case Report.” Journal of Neurosurgery 44: 92–95. 1059735 10.3171/jns.1976.44.1.0092

[vms370771-bib-0021] Kuntz, C. A. , W. S. Dernell , B. E. Powers , and S. Withrow . 1998. “Extraskeletal Osteosarcomas in Dogs: 14 Cases.” Journal of the American Animal Hospital Association 34: 26–30. 9527426 10.5326/15473317-34-1-26

[vms370771-bib-0022] Lam, R. M. Y. , G. M. Malik , and J. L. Chason . 1981. “Osteosarcoma of Meninges: Clinical, Light, and Ultrastructural Observations of a Case.” American Journal of Surgical Pathology 5: 203–208. 6939339

[vms370771-bib-0023] Langenbach, A. , M. A. Anderson , D. M. Dambach , K. U. Sorenmo , and F. D. Shofer . 1998. “Extraskeletal Osteosarcomas in Dogs: A Retrospective Study of 169 Cases (1986‐1996).” Journal of the American Animal Hospital Association 34: 113–120. 9507423 10.5326/15473317-34-2-113

[vms370771-bib-0024] Lean, F. Z. X. , B. J. Haythornthwaite , S. E. Broughton , et al. 2025. “Primary Extraskeletal Duodenal Osteosarcoma With Peritoneal Sarcomatosis in a Cat.” Journal of Comparative Pathology 219: 1–5. 40233452 10.1016/j.jcpa.2025.03.002

[vms370771-bib-0025] Liu, S.‐K. , H. D. Dorfman , and A. K. Patnaik . 1974. “Primary and Secondary Bone Tumours in the Cat.” Journal of Small Animal Practice 15: 141–156. 4532161 10.1111/j.1748-5827.1974.tb05671.x

[vms370771-bib-0026] Negrin, A. , M. Bernardini , A. Diana , and M. Castagnaro . 2006. “Giant Cell Osteosarcoma in the Calvarium of a Cat.” Veterinary Pathology 43: 179–182. 16537935 10.1354/vp.43-2-179

[vms370771-bib-0027] Norris, C. R. , S. M. Griffey , and V. F. Samii . 1999. “Pulmonary Thromboembolism in Cats: 29 Cases (1987–1997).” Journal of the American Veterinary Medical Association 215: 1650–1654. 14567429

[vms370771-bib-0028] Ohara, N. , K. Hayashi , C. Shinohara , et al. 1994. “Primary Osteosarcoma of the Cerebrum With Immunohistochemical and Ultrastructural Studies: Report of a Case.” Acta Neuropathologica 88: 384–388. 7839833 10.1007/BF00310384

[vms370771-bib-0029] O'Neill, D. G. , G. L. Edmunds , J. Urquhart‐Gilmore , et al. 2023. “Dog Breeds and Conformations Predisposed to Osteosarcoma in the UK: A VetCompass Study.” Canine Medicine and Genetics 10: 8. 37365662 10.1186/s40575-023-00131-2 PMC10294386

[vms370771-bib-0030] Osipov, V. , K.‐C. Ho , H. G. Krouwer , G. Meyer , and V. B. Shidham . 2002. “Post‐Radiation Dedifferentiation of Meningioma Into Osteosarcoma.” BMC Cancer 2: 34. 12464160 10.1186/1471-2407-2-34 PMC138810

[vms370771-bib-0031] Pace, V. , E. Persohn , and K. Heider . 1995. “Spontaneous Osteosarcoma of the Meninges in an Albino Rat.” Veterinary Pathology 32: 204–207. 7771066 10.1177/030098589503200219

[vms370771-bib-0032] Pal, P. , J. P. Holland , A. Herwadakar , and D. G. du Plessis . 2008. “Neoplasia as a Late and Rare Complication of Contrast Agent Instillation in the CNS: II‐Primary Osteosarcoma of the Spinal Meninges Arising From Post‐Arachnoiditis Osseous Metaplasia.” Neuropathology and Applied Neurobiology 34: 19.

[vms370771-bib-0033] Patnaik, A. K. 1990. “Canine Extraskeletal Osteosarcoma and Chondrosarcoma: A Clinicopathologic Study of 14 Cases.” Veterinary Pathology 27: 46–55. 2309381 10.1177/030098589002700107

[vms370771-bib-0034] Peiffer, R. L. , T. Monticello , and T. W. Bouldin . 1988. “Primary Ocular Sarcomas in the Cat.” Journal of Small Animal Practice 29: 105–116.

[vms370771-bib-0035] Pérez‐Accino, J. , A. Suñol , E. Munro , A. W. Philbey , and K. Marioni‐Henry . 2019. “Feline Meningioma With Extensive Nasal Involvement.” Journal of Feline Medicine and Surgery Open Reports 5: 2055116919833732. 30834133 10.1177/2055116919833732 PMC6393824

[vms370771-bib-0036] Pourrashidi Boshrabadi, A. , M. Surakiazad , K. K. Yarandi , and A. Amirjamshidi . 2017. “Primary Intraventricular Osteosarcoma in a 3‐Year‐Old Boy: Report of a Case and Review of Literature.” Child's Nervous System 33: 1389–1394. 10.1007/s00381-017-3450-x 28623518

[vms370771-bib-0037] Qiu, Y. Q. , and Y. L. Chen . 2021. “Primary Meningeal Osteoblastic Osteosarcoma Containing Fibroblast Osteosarcoma: Clinicopathological Analysis and Literature Review.” Osteoporosis International 32: 1007–1012. 33047193 10.1007/s00198-020-05675-8

[vms370771-bib-0038] Quigley, P. J. , and A. H. Leedale . 1983. “Tumors Involving Bone in the Domestic Cat: A Review of Fifty‐Eight Cases.” Veterinary Pathology 20: 670–686. 6580773 10.1177/030098588302000603

[vms370771-bib-0039] Reznik, M. , and J. Lenelle . 1991. “Primary Intracerebral Osteosarcoma.” Cancer 68: 793–797. 1855179 10.1002/1097-0142(19910815)68:4<793::aid-cncr2820680422>3.0.co;2-u

[vms370771-bib-0040] Riggers, D. S. , M. Rosati , C. Köhler , K. Matiasek , and S. Loderstedt . 2022. “A Case of Extraosseous Intradural Osteosarcoma of the Spine in a Dog.” Veterinary Record Case Reports 10: e470.

[vms370771-bib-0041] Ringenberg, M. A. , L. E. Neitzel , and J. F. Zachary . 2000. “Meningeal Osteosarcoma in a Dog.” Veterinary Pathology 37: 653–655. 11105956 10.1354/vp.37-6-653

[vms370771-bib-0042] Rissi, D. R. 2023. “A Review of Primary Central Nervous System Neoplasms of Cats.” Veterinary Pathology 60: 294–307. 36803009 10.1177/03009858231155400

[vms370771-bib-0043] Romeo, E. , O. Gisserot , J. P. De Jaureguiberry , et al. 2010. “Meningeal Chondroblastic Osteosarcoma: Case Report and Review of the Literature.” Journal of Neuro‐Oncology 100: 305–309. 20431908 10.1007/s11060-010-0167-z

[vms370771-bib-0044] Rosolem, M. C. , M. G. Laranjeira , R. Costa , et al. 2021. “Meningeal Osteosarcoma in a Dog's Brain.” Veterinária Notícias 27: 24–33.

[vms370771-bib-0045] Saesue, P. , E. Chankaew , O. Chawalparit , N. Sudasna Na Ayudhya , S. Muangsomboon , and T. Sangruchi . 2004. “Primary Extraskeletal Osteosarcoma in the Pineal Region: Case Report.” Journal of Neurosurgery 101: 1061–1064. 15597771 10.3171/jns.2004.101.6.1061

[vms370771-bib-0046] Sanno, N. , S. Hayashi , T. Shimura , S. Maeda , and A. Teramoto . 2004. “Intracranial Osteosarcoma After Radiosurgery—Case Report.” Neurologia Medico‐Chirurgica 44: 29–32. 14959934 10.2176/nmc.44.29

[vms370771-bib-0047] Sarmiento, J. , I. Ferrer , L. Pons , and E. Ferrer . 1979. “Cerebral Mixed Tumour: Osteo‐Condrosarcoma—Glioblastoma Multiforme: Report of One Case.” Acta Neurochirurgica 50: 335–341. 229700 10.1007/BF01808532

[vms370771-bib-0048] Saunders, J. H. , and S. A. Boroffka . 2018. “Non‐Neurologic Conditions of the Head and Neck.” In Diagnostic MRI in Dogs and Cats, edited by M. Wilfried , 393–410. CRC Press.

[vms370771-bib-0049] Schiller, M. D. , R. J. Mobbs , and S. F. Bonar . 2013. “A Case of Intradural Osteosarcoma of the Spine.” Spine Journal 13: e55–e58. 10.1016/j.spinee.2013.01.051 23578988

[vms370771-bib-0050] Sell, S. , C. Gaissmaier , J. Fritz , et al. 1998. “Different Behavior of Human Osteoblast‐Like Cells Isolated From Normal and Heterotopic Bone In Vitro.” Calcified Tissue International 62: 51–59. 9405734 10.1007/s002239900394

[vms370771-bib-0051] Setzer, M. , J. Lang , B. Turowski , and G. Marquardt . 2002. “Primary Meningeal Osteosarcoma: Case Report and Review of the Literature.” Neurosurgery 51: 488–492. 12182789

[vms370771-bib-0052] Sipos, E. P. , R. J. Tamargo , J. I. Epstein , and R. B. North . 1997. “Primary Intracerebral Small‐Cell Osteosarcoma in an Adolescent Girl: Report of a Case.” Journal of Neuro‐Oncology 32: 169–174. 9120547 10.1023/a:1005775818317

[vms370771-bib-0053] Stimson, E. L. , W. T. Cook , M. M. Smith , S. D. Forrester , M. L. Moon , and G. K. Saunders . 2000. “Extraskeletal Osteosarcoma in the Duodenum of a Cat.” Journal of the American Animal Hospital Association 36: 332–336. 10914533 10.5326/15473317-36-4-332

[vms370771-bib-0054] Tam, C. , S. Hecht , W. Mai , N. Nelson , A. V. Chen , and J. F. Griffin . 2022. “Cranial and Vertebral Osteosarcoma Commonly Has T2 Signal Heterogeneity, Contrast Enhancement, and Osteolysis on MRI: A Case Series of 35 Dogs.” Veterinary Radiology & Ultrasound: The Official Journal of the American College of Veterinary Radiology and the International Veterinary Radiology Association 63: 552–562. 35452145 10.1111/vru.13093

[vms370771-bib-0055] Troxel, M. T. , C. H. Vite , C. Massicotte , et al. 2004. “Magnetic Resonance Imaging Features of Feline Intracranial Neoplasia: Retrospective Analysis of 46 Cats.” Journal of Veterinary Internal Medicine 18: 176–189. 15058768 10.1892/0891-6640(2004)18<176:mrifof>2.0.co;2

[vms370771-bib-0056] Troxel, M. T. , C. H. Vite , T. J. Van Winkle , et al. 2003. “Feline Intracranial Neoplasia: Retrospective Review of 160 Cases (1985–2001).” Journal of Veterinary Internal Medicine 17: 850–859. 14658723 10.1111/j.1939-1676.2003.tb02525.x

[vms370771-bib-0057] Tsantes, A. G. , I. Loukopoulou , D. V. Papadopoulos , et al. 2022. “The Hypercoagulable Profile of Patients With Bone Tumors: A Pilot Observational Study Using Rotational Thromboelastometry.” Cancers 14: 3930. 36010924 10.3390/cancers14163930 PMC9406421

[vms370771-bib-0058] Turner, O. A. , and W. M. Craig . 1941. “Osteogenic Sarcoma of Meningeal Origin.” Archives of Pathology 32: 103–111.

[vms370771-bib-0059] Walker, M. T. , L. R. Toye , S. W. Coons , R. W. Porter , and R. C. Wallace . 2001. “Intradural Primary Chondroblastic Osteosarcoma: Case Report.” American Journal of Neuroradiology 22: 1960–1962. 11733332 PMC7973820

[vms370771-bib-0060] Wang, A.‐M. , T. J. Fitzgerald , A. H. Lichtman , et al. 1986. “Neuroradiologic Features of Primary Falx Osteosarcoma.” American Journal of Neuroradiology 7: 729–732. 3088951 PMC8334652

[vms370771-bib-0061] Woog, J. , D. M. Albert , J. R. Gonder , and J. J. Carpenter . 1983. “Osteosarcoma in a Phthisical Feline Eye.” Veterinary Pathology 20: 209–214. 6573055 10.1177/030098588302000208

[vms370771-bib-0062] Zhang, S. , Y. Ju , and C. You . 2016. “A Rare Case of Extensive Primary Meningeal Osteosarcoma in Childhood.” Neurology 87: 1420–1421. 27672166 10.1212/WNL.0000000000003159

[vms370771-bib-0063] Ziewacz, J. , J. Song , M. Blaivas , and L. J. S. Yang . 2010. “Radiation‐Induced Meningeal Osteosarcoma of Tentorium Cerebelli With Intradural Spinal Metastases.” Surgical Neurology International 1: 14. 20657695 10.4103/2152-7806.63909 PMC2908355

[vms370771-bib-0064] Zirh, T. A. , M. N. Pamir , M. M. Ozek , C. Erzen , and A. Sav . 1992. “Primary Osteogenic Sarcoma of the Falx Cerebri: A Case Report.” European Journal of Radiology 15: 193–195. 1490442 10.1016/0720-048x(92)90104-h

